# An Assessment of In Vitro Antifungal Activities of Efinaconazole and Itraconazole against Common Non-Dermatophyte Fungi Causing Onychomycosis

**DOI:** 10.3390/jof3020020

**Published:** 2017-05-05

**Authors:** Ananya Tupaki-Sreepurna, Bhavna T. Jishnu, Vijayakishore Thanneru, Savitri Sharma, Anjana Gopi, Murugan Sundaram, Anupma Jyoti Kindo

**Affiliations:** 1 Department of Microbiology, Sri Ramachandra Medical College and Research Institute, Sri Ramachandra University, Porur, Chennai Tamilnadu 600116, India; ts.ananya@gmail.com (A.T.-S.); bhavimagesh@gmail.com (B.T.J.); tvk.msc@gmail.com (V.T.); 2 Jhaveri Microbiology Centre, LV Prasad Eye Institute, Kallam Anji Reddy Campus, L V Prasad Marg, Banjara Hills, Hyderabad Telangana 500034, India; savitri@lvpei.org; 3 Department of Microbiology, Kempegowda Institute of Medical Sciences, Athibabbe Road, Banashankari Stage II, Banashankari, Bengaluru Karnataka 560070, India; dranjanagopi1989@gmail.com; 4 Department of Dermatology, Venereology & Leprosy, Sri Ramachandra Medical College and Research Institute, Sri Ramachandra University, Porur, Chennai Tamilnadu 600116, India; murugan1972@gmail.com

**Keywords:** antifungal susceptibility testing, *Fusarium*, *Aspergillus*, CLSI, efinaconazole, itraconazole, onychomycosis

## Abstract

Onychomycosis is a fungal nail infection which is relatively common and difficult to treat. Treatment modalities include nail avulsion, surgical debridement and combination therapy with oral and topical antifungal drugs. In spite of a host of available drugs, clinical cure rates remain discouraging. Drug toxicities, prolonged regimens, lack of patient compliance, and high keratin affinity of drugs are all contributive factors. Efinaconazole is a novel topical triazole antifungal agent that has shown excellent in vitro activity against both dermatophyte and non-dermatophyte fungi causing onychomycosis. This study presents the in vitro susceptibility profiles of 44 common non-dermatophyte fungi against efinaconazole and itraconazole, another azole drug used in the treatment of onychomycosis.

## 1. Introduction

Onychomycosis, a fungal nail infection affecting either the toenails (more common) or fingernails, involving any or all components of the nail structure, is typically chronic and tough to treat. When the causative agent is a dermatophyte, the infection is termed tinea unguium. Common symptoms are thickening of the nails with discoloration, brittleness or deformity and prolonged infection may be accompanied by pain under the nail and while wearing shoes, due to nail thickening or hyperkeratosis. People with these infections have been found to suffer psychosocial stress due to the cosmetic repercussions of an unhealing infection. Tinea unguium due to dermatophytes (*Trichophyton*, *Epidermophyton* species) occurs more often in temperate regions, whereas onychomycosis due to non-dermatophyte moulds and yeasts like *Candida* and *Trichosporon* species are seen more commonly in the tropical regions of the world. Among the non-dermatophyte moulds, the common onychomycosis-causing fungi are *Scytalidium* (or *Neoscytalidium*), *Aspergillus*, *Fusarium*, *Scopulariopsis*, *Paecilomyces*, *Pseudoallescheria*, *Penicillium* and *Alternaria* species. Rarer species implicated are *Onychocola* species, *Pyrenochaeta* species, *Chaetomium globosum* and *Lasiodiplodia theobromae*. All fungi involved exhibit keratinolytic activity [1,2,3]. Some studies have stressed the significant prevalence of non-dermatophyte fungi (NDF) in onychomycosis. A 2015 study showed non-dermatophyte moulds emerging as a leading cause of onychomycosis in south-east Rajasthan in India [4]. In 2016, a growing incidence of non-dermatophyte onychomycosis was reported from Tehran, Iran with *Aspergillus* as the most common cause, followed by *Fusarium* [5]. NDF have also been seen to colonize damaged nail plates [6].

Predisposing risk factors to onychomycosis include trauma, frequent contact with water, lack of foot hygiene, tinea pedis, immunodeficiency, genetic predisposition, psoriasis, diabetes mellitus and old age (nail ischaemia). Traditionally, onychomycosis has been classified as distal subungual onychomycosis, proximal subungual onychomycosis, white superficial onychomycosis and *Candida* infections of the nail, while end-stage nail disease is termed total dystrophic onychomycosis [1,2,3]. In 2011, the Onychomycosis Severity Index (OSI) was created, a simple grading and prognostic tool showing high consistency and reliability, in which non-dermatophyte moulds and yeasts as causative agents were included as one of the factors indicating poor prognosis [7].

Currently, the preferred drugs for treating onychomycosis are oral itraconazole and terbinafine, in combination with topical nail lacquers such as ciclopirox and amorolfine [8]. In spite of the combination therapy, cure rates are considerably low. Factors contributing to ineffective treatment may include the existing hyperkeratosis in the infected nail that may limit drug penetration. In addition, prolonged treatment regimens of six to twelve months may deter the patient from being compliant, especially when the recurrence rates after apparent cure are quite high. Furthermore, the development of antifungal resistance is an emerging concern. Terbinafine resistance mechanisms have now been identified in *Trichophyton rubrum*, *Aspergillus nidulans* and *Aspergillus fumigatus* [9,10,11]. Most of the anti-onychomycosis drugs target the pathway of fungal ergosterol biosynthesis, an essential structural component of the fungal cell membrane. Itraconazole, like all azole antifungals, inhibits lanosterol 14α-demethylase, an enzyme of the cytochrome P450-type. Caution is warranted due to possible drug–drug interactions, especially in the elderly who may be on many medications simultaneously. Possible hepatotoxicity requires monitoring of liver enzymes during the treatment period. Itraconazole differs from the other azole drugs, because in addition to the above, its mechanism of action includes inhibition of the hedgehog signaling pathway [12] and angiogenesis, giving it anticancer properties as well as antifungal. Terbinafine exhibits fungicidal activity by potent non-competitive inhibition of squalene epoxidase enzyme, leading to the accumulation of intracellular squalene and decreased ergosterol. As squalene epoxidase is not an enzyme of the cytochrome P450-type, this class of enzymes is not inhibited and cholesterol biosynthesis in vivo is also unaffected, leading to lesser toxicity associated with prolonged treatment. Notably however, liver toxicity has been occasionally linked to Terbinafine [13]. The mechanism of action of topical ciclopirox is believed to involve its high affinity to trivalent metal cations, leading to the inhibition of essential co-factors to catalase and peroxidase enzymes and resulting in the loss of enzyme function. Amorolfine is a morpholine topical antifungal drug that interferes with ergosterol biosynthesis at two steps, leading to the accumulation of an intermediate compound in the cell membrane while ergosterol is depleted [1,2,3,8].

To be effective against onychomycosis, adequate amounts of all the aforementioned drugs must penetrate the nail bed and remain active within the keratin matrix of the nail and nail bed. Keratin-bound drugs have been found to have decreased penetration, resulting in drug accumulation in surface nail layers with little or no amounts in the deeper layers and nail bed. A low affinity to keratin is desirable when choosing the ideal drug to treat onychomycosis. Efinaconazole is a relatively new antifungal synthesized as an azole amine derivative by Kaken Pharmaceutical Co. The drug was approved and launched in Canada (2013), the US and Japan (2014) for the topical treatment of onychomycosis. Formulated as a 10% solution containing 100 mg of efinaconazole per gram in a clear, colorless to pale yellow solution [14], it acts by inhibiting lanosterol 14α demethylase and its in vitro activity was found to be minimally affected by keratin. The methylene-piperidine group at the C-4 position of its molecular structure may be responsible for its low keratin binding. High nail penetration and potent fungicidal activity in the presence of keratin were observed both in vitro and in vivo. This property makes it almost ideal for the treatment of onychomycosis. It has low surface tension, which aids in penetration and spreading, and is sparingly soluble in water. The drug accesses the site of infection by both transungual delivery and spreading through the subungual space [15,16,17,18,19]. In a guinea pig onychomycosis model, topically applied efinaconazole was found to be more effective in reducing toenail fungal burden than were amorolfine and terbinafine [20]. Efinaconazole has also been shown to possess a broader spectrum of activity than existing antifungals against dermatophyte and non-dermatophyte moulds and yeasts, including *Fusarium* species, which cause nail infections that respond poorly to oral drugs [21,22,23].

In the present study, we tested the in vitro activities of two drugs, itraconazole and efinaconazole, against 44 isolates of the most common non-dermatophyte fungi causing onychomycosis.

## 2. Materials and Methods

### 2.1. Study Isolates

Forty-four clinical isolates of non-dermatophyte fungi commonly causing onychomycosis were collected from Sri Ramachandra Medical College and Research Institute (Chennai, India), LV Prasad Eye Institute (Hyderabad, India) and Kempegowda Institute of Medical Sciences (Bengaluru, India) and used for in vitro susceptibility testing against itraconazole and efinaconazole. In total, 12 *F. falciforme*, 4 *F. keratoplasticum*, 2 unnamed members of the *Fusarium solani* species complex, 2 *F. delphinoides*, 1 *F. incarnatum*, 10 *A. flavus*, 2 *A. terreus*, 5 *A. niger*, 3 *A. fumigatus*, 1 *Alternaria* species, 1 *Penicillium* species, and 1 *Candida albicans* were tested. The isolates were cultured on Sabouraud Dextrose Agar (SDA), Potato Dextrose Agar (PDA) and Oatmeal Agar (OA) (HiMedia Laboratories Pvt. Ltd., Mumbai, India). Macroscopic observations including growth rate, and features of colony morphology such as texture, colour and production of diffusible pigment were noted. The microscopic features like conidia formation, presence of macroconidia (with number/nature of septa), presence of microconidia and chlamydospores were observed in lactophenol cotton blue wet mounts. Speciation was done accordingly. For *Fusarium* isolates, speciation was done by DNA sequencing of TEF-1α (Translation Elongation Factor 1-alpha) using primers EF1 (forward primer; 5′-ATGGGTAAGGA(A/G)GACAAGAC-3′) and EF2 (reverse primer; 5′-GGA(G/A)GTACCAGT(G/C)ATCATGTT-3′) [24] and BLAST (Basic Local Alignment and Search Tool) matching at NCBI GenBank (http://www.ncbi.nlm.nih.gov/BLAST/) and Fusarium-ID (http://isolate.fusariumdb.org/index.php) databases.

### 2.2. Antifungal Agents

Antifungal drugs were obtained in pure powder form; itraconazole (Sigma-Aldrich, St. Louis, MO, USA) and efinaconazole (Hwasun Technologies, Shanghai, China) and were dissolved in dimethyl sulfoxide (DMSO) (Sigma-Aldrich, St. Louis, MO, USA) to make stock solutions of concentrations 100X the highest concentration to be tested. Itraconazole was tested in the range of 0.06125–32 µg/mL for *Fusarium* species and in the range of 0.0078–4 µg/mL for *Aspergillus* and other species. Efinaconazole was tested in the range of 0.0078–4 µg/mL for all isolates.

### 2.3. In Vitro Antifungal Susceptibility Testing

Antifungal susceptibility testing was done according to the CLSI-M38-A2 guidelines following the broth microdilution method described by the Clinical and Laboratory Standards Institute (CLSI) [25]. The fungal growth medium used was the synthetic broth medium RPMI-1640 with L-glutamine, without sodium bicarbonate and with phenol red as a pH indicator (HiMedia Laboratories, Mumbai, India) buffered to a pH of 7.0 at 25 °C using MOPS (3-[*N*-morpholino] propanesulfonic acid) buffer (Sigma-Aldrich, St. Louis, MO, USA). A saline spore suspension of a ten-day-old well-sporulated culture on Potato Dextrose Agar (PDA) slant (HiMedia Laboratories Pvt. Ltd., Mumbai, India) was used for inoculum preparation. With the turbidity of the saline spore suspension adjusted to 0.5 McFarland Turbidity Standard (Appendix C section of CLSI M38-A2)., it was then further diluted 1:50 times (for moulds) and 1:500 (for yeasts) in RPMI-1640 with l-glutamine and without sodium bicarbonate This served as the final inoculum and 0.1 mL i.e., 100 µL of this inoculum suspension and 100 µL of the antifungal drug dilutions were added to the wells of the microdilution plates. Each drug was designated one row in the plate with each well in the row for a particular drug concentration. The growth control well contained 100 µL of the inoculum and 100 µL of the drug diluent without antifungal agent. Sterility control was also included with plain RPMI-1640 medium without inoculum. *Paecilomyces variotii* CBS 132734 was included in the tests as a quality control strain. The plates were incubated at 35 °C and the readings were taken after 48 h of incubation. The first well showing 100% inhibition of growth in the case of moulds and 50% inhibition of growth in the case of yeasts were taken as the Minimum Inhibitory Concentration (MIC). Susceptibility testing was performed in triplicate and the modal MIC for each drug was selected as the MIC value of the drug for the particular isolate.

### 2.4. Statistical Analysis

All MIC data for efinaconazole and itraconazole were recorded and analyzed using Microsoft Excel 2010. Geometric mean MIC, MIC50, MIC90 and cumulative MIC frequency of both drugs were calculated.

## 3. Results

### 3.1. Susceptibility Pattern of Test Isolates against Efinaconazole and Itraconazole

Table 1 concisely represents the MIC range and geometric mean MIC of all 44 test isolates of the present study.

### 3.2. Fusarium Species

**Figure 1:**
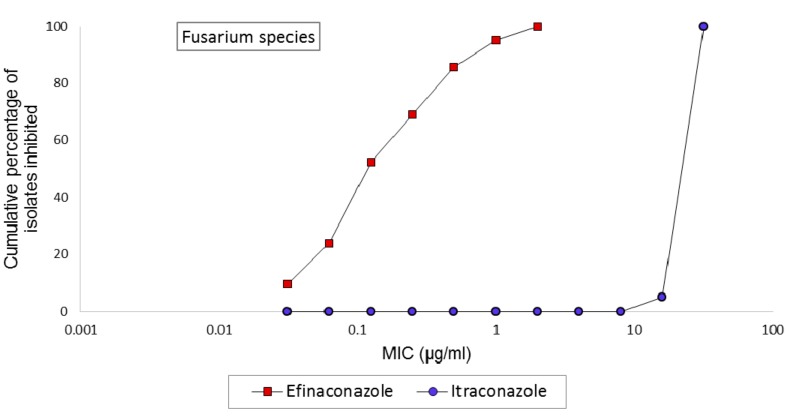
Cumulative MIC frequency distribution of efinaconazole and itraconazole against clinical *Fusarium* species (*n* = 21).

Efinaconazole inhibited fungal growth at concentrations ranging from 0.03125–2 µg/mL (Table 1). However, based on the MIC90 for the genus (not shown in Table 1), most of the *Fusarium* isolates were inhibited between 0.25–0.5 µg/mL. *Fusarium dimerum* species complex showed the least MIC for efinaconazole (geometric mean MIC 0.088 µg/mL). The cumulative MIC frequency of efinaconazole against 21 *Fusarium* species tested is shown in Figure 1. In contrast, itraconazole failed to exhibit inhibitory action against *Fusarium* with high MICs 16–>32 µg/mL. There were no differences in susceptibility patterns by geographical location.

### 3.3. Aspergillus Species

**Figure 2:**
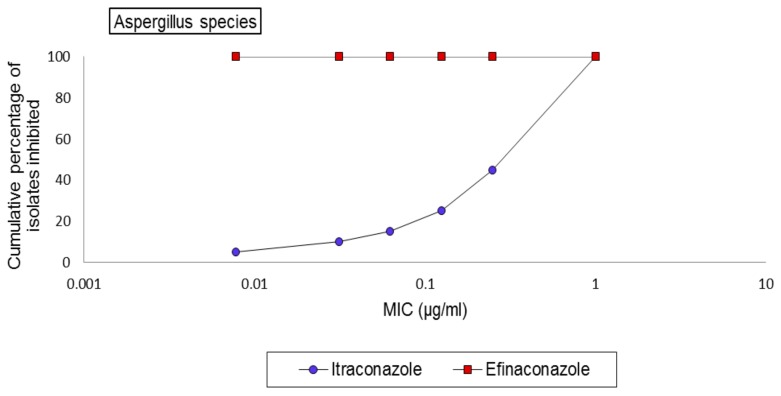
Cumulative MIC frequency distribution of efinaconazole and itraconazole against clinical *Aspergillus* species (*n* = 20).

Efinaconazole showed uniformly excellent activity against all *Aspergillus* species tested, with an MIC equal to 0.0078 µg/mL, the lowest concentration of the drug tested. Itraconazole also showed good activity against *Aspergillus* with an MIC range of 0.0078–1 µg/mL, with most isolates being inhibited between 0.25–1 µg/mL, based on the MIC90 data for the genus (not shown in Table 1). *Aspergillus flavus* showed the maximum susceptibility to itraconazole among the *Aspergillus* species, with a geometric mean MIC of 0.22 µg/mL. The cumulative MIC frequency of itraconazole against 20 *Aspergillus* species tested is shown (Figure 2). There were no differences in susceptibility patterns by geographical location.

### 3.4. Other Species

Three isolates, one each of *Penicillium* sp., *Alternaria* sp. and *Candida albicans* that were tested showed an MIC of 0.016 µg/mL for efinaconazole and and MIC of 0.25 µg/mL (*Alternaria* sp.) and 0.125 µg/mL (*Candida albicans* and *Penicillium* sp.) for itraconazole.

## 4. Discussion

In the present study, itraconazole MICs of test isolates were in the range as established by previous studies [21,22,26,27,28]. MICs of efinaconazole were significantly lower than that of itraconazole in all 44 isolates tested (*p* < 0.001, unpaired *t*-test). Especially for *Fusarium* species, which are known to cause recalcitrant infections [29], efinaconazole showed roughly eight-fold more activity than itraconazole based on the geometric mean MICs of both the drugs. Itraconazole MICs were above the epidemiological cut-off values (ECVs) recently established for *Fusarium* [30]. A few researchers have also reported similar findings regarding *Fusarium* MICs to efinaconazole [23]. In other studies [16], two- to three-fold greater mycological and complete cure rates were observed clinically with efinaconazole 10% topical solution than those with other topical antifungal drugs. Based on geometric mean MICs, efinaconazole showed 74-fold more activity than itraconazole for *Aspergillus* species in the present study, comparable to other studies [23]. From the MIC data, efinaconazole is seen to be more potent than itraconazole against different non-dermatophyte fungi causing onychomycosis and can be recommended as an effective drug for use in treatment. Early treatment of onychomycosis to avoid disease progression to other toenails is important. An infected nail also serves as a mycotic reservoir and a potential portal of entry for systemic dissemination [1,2,3].

The low keratin affinity of efinaconazole contributes to the drug’s nail penetration and fungicidal activity in onychomycosis treatment [16]. The morphological changes seen in fungal structure due to exposure to efinaconazole include shortening of interseptal distance, globular swellings, non-uniform widths and flattening. The ultrastructural changes consist of thickening of the cell wall, separation of plasma membrane from the cell wall, accumulation of electron-dense granules in the space between the cell wall and plasma membrane, and discontinuity of the plasma membrane along with degeneration of intracytoplasmic organelles. All these changes are likely to be responsible for cell death [15].

Prolonged antifungal drug exposure can induce a stress response in fungal cells, leading to a decrease in drug susceptibility via mechanisms such as the induction of drug efflux pumps. However, a study by Iwata et al. [31] observed no evidence of efinaconazole resistance under experimental conditions among dermatophytes, suggesting that efinaconazole has low potential to induce drug resistance, at least among dermatophyte species.

Efinaconazole 10% topical solution marketed as Jublia (the US and Canada) and Clenafin (Japan) has a better safety profile than oral itraconazole therapy which requires blood monitoring [32]. It is labeled pregnancy class C with a caution from the FDA (Food and Drug Administration) regarding use in breastfeeding women, although it is unknown if it is excreted into human milk. The typical duration of treatment is an application once daily with a flow-through brush applicator for 48 weeks. As the drug is topical, patient compliance is better and associated toxicities are lesser than those with oral itraconazole. Many cases of onychomycosis are of mixed-pathogen nature, and the broad spectrum of activity of efinaconazole is useful in treating such cases. Unfortunately, efinaconazole is still not available in the Indian market [33]. Thousands of patients could benefit from this drug if it is made available in India at the earliest.

## 5. Conclusions

Efinaconazole proves to be a more potent drug than itraconazole for the treatment of onychomycosis due to non-dermatophyte fungi including *Fusarium* species. Its topical route of administration and low keratin binding make it more suitable for prolonged treatment than the oral drugs currently in use. Efinaconazole needs to be made available for patients in India.

## Figures and Tables

**Table 1:** In vitro antifungal activities of efinaconazole and itraconazole against common non-dermatophyte fungal agents of onychomycosis.

Organism (Number of Isolates)	Drug	MIC (µg/mL)	
		Range	Geometric Mean
*Fusarium falciforme* (12)	Efinaconazole	0.063–1	0.19
	Itraconazole	>32	>32
*Fusarium keratoplasticum* (4)	Efinaconazole	0.032–2	0.18
	Itraconazole	>32	>32
*Fusarium solani* species complex — unnamed members (2)	Efinaconazole	0.50–1	0.70
	Itraconazole	>32	>32
*Fusarium dimerum* species complex (2)	Efinaconazole	0.032–0.25	0.088
	Itraconazole	16–32	22.63
*Fusarium incarnatum equiseti* species complex (1)	Efinaconazole	0.50	0.50
	Itraconazole	>32	>32
*Aspergillus flavus* (10)	Efinaconazole	0.0078	0.0078
	Itraconazole	0.0078–1	0.22
*Aspergillus niger* (5)	Efinaconazole	0.0078	0.0078
	Itraconazole	0.25–1	0.57
*Aspergillus fumigatus* (3)	Efinaconazole	0.0078	0.0078
	Itraconazole	1	1
*Aspergillus terreus* (2)	Efinaconazole	0.0078	0.0078
	Itraconazole	0.25	0.25
*Penicillium* species (1)	Efinaconazole	0.016	0.016
	Itraconazole	0.13	0.13
*Alternaria* species (1)	Efinaconazole	0.016	0.016
	Itraconazole	0.25	0.25
*Candida albicans* (1)	Efinaconazole	0.016	0.016
	Itraconazole	0.13	0.13

## References

[1] Elewski B.E. (1998). Onychomycosis: Pathogenesis, Diagnosis and management. Clin. Microbiol. Rev..

[2] Summerbell R.C., Kane J., Krajden S. (1989). Onychomycosis, tinea pedis and tinea manuum caused by non-dermatophytic filamentous fungi. Mycoses.

[3] Welsh O., Vera-Cabrera L., Welsh E. (2010). Onychomycosis. Clin. Dermatol..

[4] Raghavendra K.R., Yadav D., Kumar A., Sharma M., Bhuria J., Chand A.E. (2015). The nondermatophyte molds: Emerging as leading cause of onychomycosis in south-east Rajasthan. Indian Dermatol. Online J..

[5] Motamedi M., Ghasemi Z., Shidfar M.R., Hosseinpour L., Khodadadi H., Zomorodian K., Mirhendi H. (2016). Growing incidence of non-dermatophyte onychomycosis in Tehran, Iran. Jundishapur J. Microbiol..

[6] El Batawi M.M., Arnaot H., Shoeib S., Bosseila M., Fangary M.E., Helmy A.S. (2006). Prevalence of non-dermatophyte molds in patients with abnormal nails. Egypt. Dermatol. Online J..

[7] Carney C., Tosti A., Daniel R., Scher R., Rich P., DeCoster J., Elewski B. (2011). A new classification system for grading the severity of onychomycosis: Onychomycosis Severity Index. Arch. Dermatol..

[8] Gupta A.K., Paquet M., Simpson F.C. (2013). Therapies for the treatment of onychomycosis. Clin. Dermatol..

[9] Martinez-Rossi N.M., Peres N.T.A., Rossi A. (2008). Antifungal resistance mechanisms in dermatophytes. Mycopathologia.

[10] Rocha E.M.F., Gardiner R.E., Park S., Martinez-Rossi N.M., Perlin D.S. (2006). A Phe389Leu substitution in ErgA confers terbinafine resistance in *Aspergillus fumigatus*. Antimicrob. Agents Chemother..

[11] Mukherjee P.K., Leidich S.D., Isham N., Leitner I., Ryder N.S., Ghannoum M.A. (2003). Clinical *Trichophyton rubrum* strain exhibiting primary resistance to terbinafine. Antimicrob. Agents Chemother..

[12] Kim J., Tang J.Y., Gong R., Kim J., Lee J.J., Clemons K.V., Chong C.R., Chang K.S., Fereshteh M., Gardner D. (2010). Itraconazole, a commonly used antifungal that inhibits hedgehog pathway activity and cancer growth. Cancer Cell..

[13] Ajit C., Zaeri N., Munoz S.J., Suvannasankha A. (2003). Terbinafine–associated hepatotoxicity. Am. J. Med. Sci..

[14] Lipner S.R., Scher R.K. (2015). Efinaconazole in the treatment of onychomycosis. Infect. Drug Resist..

[15] Tatsumi Y., Nagashima M., Shibanushi T., Iwata A., Kangawa Y., Inui F., Siu W.J.J., Pillai R., Nishiyama Y. (2013). Mechanism of action of efinaconazole, a novel triazole antifungal agent. Antimicrob. Agents Chemother..

[16] Sugiura K., Sugimoto N., Hosaka S., Katafuchi-Nagashima M., Arakawa Y., Tatsumi Y., Siu W.J.J., Pillai R. (2014). The low keratin affinity of efinaconazole contributes to its nail penetration and fungicidal activity in topical onychomycosis treatment. Antimicrob. Agents Chemother..

[17] Rich P. (2015). Efinaconazole topical solution, 10%: The benefits of treating onychomycosis early. J. Drugs Dermatol..

[18] Lipner S.R., Scher R.K. (2015). Efinaconazole 10% topical solution for the topical treatment of onychomycosis of the toenail. Expert. Rev. Clin. Pharmacol..

[19] Gupta A.K., Simpson F.C. (2014). Efinaconazole (Jublia) for the treatment of onychomycosis. Expert. Rev. Anti Infect. Ther..

[20] Tatsumi Y., Yokoo M., Senda H., Kakehi K. (2002). Therapeutic efficacy of topically applied KP-103 against experimental *Tinea unguium* in guinea pigs in comparison with amorolfine and terbinafine. Atimicrob. Agents Chemother..

[21] Azor M., Gene J., Cano J., Guarro J. (2007). Universal *in vitro* antifungal resistance of genetic clades of the *Fusarium solani* species complex. Antimicrob. Agents Chemother..

[22] Alastruey-Izquierdo A., Cuenca-Estrella M., Monzon A., Meliado E., Rodriguez-Tudela J.L. (2008). Antifungal susceptibility profile of clinical *Fusarium* spp. isolates identified by molecular methods. J. Antimicrob. Chemother..

[23] Siu W.J.J., Tatsumi Y., Senda H., Pillai R., Nakamura T., Sone D., Fothergill A. (2013). Comparison of *in vitro* antifungal activities of efinaconazole and currently available antifungal agents against a variety of pathogenic fungi associated with onychomycosis. Antimicrob. Agents Chemother..

[24] Geiser D.M., Jimenez-Gasco M.D.M., Kang S., Makalowska I., Veeraraghavan N., Ward T.J., Zhang N., Kuldau G.A., O’Donnell K. (2004). Fusarium-ID v. 1.0: A DNA sequence database for identifying *Fusarium*. Eur. J. Plant Pathol..

[25] Rex J.H., Ghannoum M.A., Alexander B.D., Knapp C.C., Andes D., Motyl M.R., Arthington-Skaggs B., Ostrosky-Zeichner L., Brown S.D., Pfaller M. (2008). Reference method for broth dilution antifungal susceptibility testing of filamentous fungi. Approved standard. CLSI document M38-A2. Clin. Lab. Stand. Inst..

[26] Alastruey-Izquierdo A., Cuesta I., Ros L., Mellado E., Rodriguez-Tudela J.L. (2011). Antifungal susceptibility profile of clinical *Alternaria* spp. identified by molecular methods. J. Antimicrob. Chemother..

[27] Verweij P.E., Howard S.J., Melchers W.J.G., Denning D.W. (2009). Azole-resistance in *Aspergillus*: Proposed nomenclature and breakpoints. Drug Resist. Updates.

[28] Pfaller M.A., Boyken L., Hollis R.J., Messer S.A., Tendolkar S., Diekema D.J. (2005). In vitro susceptibilities of clinical isolates of *Candida* species, *Cryptococcus neoformans* and *Aspergillus* species to itraconazole: Global Survey of 9359 isolates tested by Clinical and Laboratory Standards Institute broth microdilution methods. J. Clin. Microbiol..

[29] Al-Hatmi A.M.S., Meis J.F., de Hoog G.S. (2016). *Fusarium*: Molecular diversity and intrinsic drug resistance. PLoS Pathog..

[30] Espinel-Ingroff A., Colombo A.L., Cordoba S., Dufresne P.J., Fuller J., Ghannoum M., Gonzalez G.M., Guarro J., Kidd S.E., Meis J.F. (2015). International evaluation of MIC distributions and epidemiological cutoff value (ECV) definitions for *Fusarium* species identified by molecular methods for the CLSI broth microdilution method. Antimicrob. Agents Chemother..

[31] Iwata A., Watanabe Y., Kumagai N., Katafuchi-Nagashima M., Sugiura K., Pillai R., Tatsumi Y. (2014). In vitro and in vivo assessment of dermatophyte acquired resistance to efinaconazole, a novel triazole antifungal. Antimicrob. Agents Chemother..

[32] Patel T., Dhillon S. (2013). Efinaconazole: First global approval. Drugs.

[33] (2015). Recent drug approvals in dermatology. Indian J. Drugs Dermatol..

